# Oxidative Stress—Related Serum Extracellular Vesicle miRNAs Indicate Symptom Severity and Cognitive Decline in Parkinson's Disease

**DOI:** 10.1111/jnc.70355

**Published:** 2026-01-18

**Authors:** Violeta Belickienė, Aistė Pranckevičienė, Andrius Radžiūnas, Andrėja Strigauskaitė, Ovidijus Laucius, Paulina Vaitkienė

**Affiliations:** ^1^ Laboratory of Molecular Neurobiology, Neuroscience Institute, Medical Academy Lithuanian University of Health Sciences Kaunas Lithuania; ^2^ Health Psychology Department, Faculty of Public Health, Medical Academy Lithuania University of Health Sciences Kaunas Lithuania; ^3^ Neuroscience Institute, Medical Academy Lithuanian University of Health Sciences Kaunas Lithuania; ^4^ Department of Neurosurgery, Medical Academy Lithuanian University of Health Sciences Kaunas Lithuania; ^5^ Medical Academy Lithuanian University of Health Sciences Kaunas Lithuania; ^6^ Neurology Department Lithuanian University of Health Sciences Kaunas Lithuania

**Keywords:** biomarker, cognition, extracellular vesicles, miRNA, neurodegeneration, Parkinson's disease

## Abstract

Parkinson's disease (PD) is a progressive neurodegenerative disorder characterized by motor and non‐motor symptoms, including cognitive decline and reduced quality of life. Identifying reliable biomarkers for disease progression and symptom severity remains a critical challenge. In this study, levels of oxidative stress–related microRNAs (miR‐24‐3p, miR‐103a‐3p, miR‐320a‐3p, miR‐494‐3p, miR‐126‐5p, and miR‐543) within blood serum extracellular vesicles (EVs) were examined in a cohort of 93 PD patients to assess their associations with cognitive function, symptom severity, quality of life, and other clinical characteristics. The methods included microRNA extraction from blood serum EVs, followed by cDNA synthesis and RT‐qPCR for expression analysis. Upregulation of miR‐126‐5p, as well as downregulation of miR‐24‐3p showed the strongest associations with symptom severity and cognitive decline, whereas downregulated miR‐320a‐3p levels correlated with patient‐reported quality of life in PD patients. Downregulation of miR‐103a‐3p, and miR‐543 expression showed slight associations with motor symptoms, cognitive function, and quality of life domains; however, some of these associations lacked statistical power. These findings indicate that specific microRNA expression profiles in extracellular vesicles are associated with PD symptom severity and progression, supporting their further evaluation as biomarkers in larger independent cohorts.

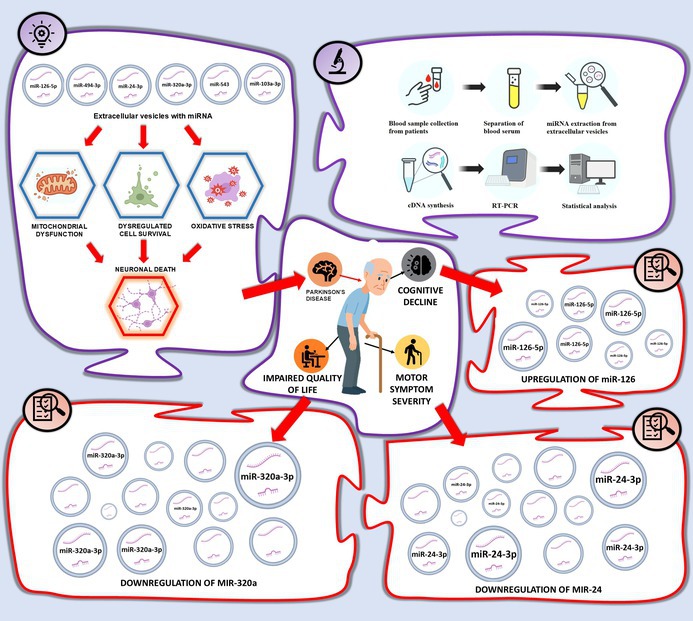

## Graphical abstract

Oxidative stress and mitochondrial dysfunction are core mechanisms to dopaminergic neuronal death in Parkinson's disease (PD) and are regulated by microRNAs such as miR‐24‐3p, miR‐103a‐3p, miR‐320a‐3p, miR‐494‐3p, miR‐126‐5p, and miR‐543. Their expression was analyzed in serum extracellular vesicles of 93 PD patients by RT‐qPCR, as well as correlations with cognition, motor symptoms, and quality of life. MiR‐126‐5p overexpression was linked with cognitive decline. Downregulation of miR‐24‐3p was correlated with motor severity, and reduced levels of miR‐320a‐3p were associated with impairment in quality of life. These findings confirm the potential of extracellular vesicle‐derived microRNAs as biomarkers for PD progression.

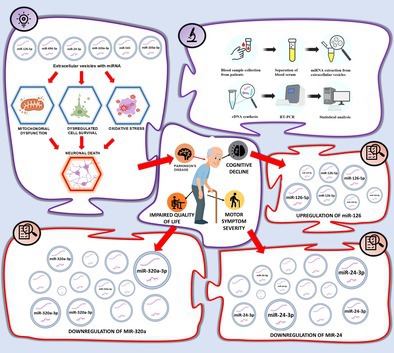

**Abbreviations**
ADL: activities of daily living
BBB: blood brain barrier
EV: extracellular vesicle
HC: healthy control
miRNA/miR: microRNA
MMSE: Mini‐Mental State Examination
MoCA: The Montreal Cognitive Assessment
ON/OFF states: motor fluctuations
PD: Parkinson disease
PDQ‐39: Parkinson's Disease Questionnaire
QOL: quality of life
RAVLT: Rey Auditory Verbal Learning Test
ROS: reactive oxygen species
RT‐qPCR: real‐time qPCR
SOD: superoxide dismutase
TMT: trail making test
UPDRS: Unified Parkinson's Disease Rating Scale
WAIS‐III: Wechsler Adult Intelligence Scale‐III

## 1 Introduction

Parkinson's disease (PD) is a progressive neurodegenerative disorder developing due to dopaminergic neuron death in the substantia nigra of the midbrain (Parkinson 2002). It is characterized by both motor symptoms, such as tremors, rigidity, and bradykinesia, and non‐motor symptoms, including cognitive decline, depression, or autonomic dysfunction, all of which significantly reduce the quality of life (QOL) (Kouli et al. 2018; Wan et al. 2023).

Increasing evidence suggests that microRNAs (miRNAs) play a crucial role in regulating gene expression and may contribute to neurodegeneration (Li et al. 2023). Specifically, miRNAs carried by extracellular vesicles (EVs) have emerged as potential biomarkers for disease progression and cognitive impairment (Chai et al. 2024). In PD, miRNAs have been found to be dysregulated in the substantia nigra, the key site of neurodegeneration (Nies et al. 2021). However, direct investigation of the living brain presents significant challenges. EVs offer a promising alternative for studying miRNA dynamics, as miRNAs are not confined to their cells of origin but can be encapsulated within EVs, cross the blood–brain barrier (BBB), and be detected in circulating blood EVs. This offers a minimally invasive approach to assess brain‐derived miRNAs, their potential role in PD pathophysiology, and provides insights into their possible therapeutic value (Belkozhayev et al. 2022).

Previous studies have shown that miR‐24‐3p (Zhou et al. 2023), miR‐103a‐3p (Zhou et al. 2020), miR‐320a‐3p (Shukla et al. 2023), miR‐494‐3p (Geng et al. 2018), miR‐126‐5p (Lee and Im 2021), and miR‐543 (Scheper et al. 2023) play critical roles in regulating key pathways of mitochondrial dysfunction and oxidative stress, which are central to PD pathogenesis (Szelągowski and Kozakiewicz 2023). For instance, miR‐103a‐3p has been shown to regulate mitophagy through Parkin/Ambra1 signaling, a crucial pathway for clearing damaged mitochondria in PD (Zhou et al. 2020). Similarly, miR‐24‐3p inhibits PINK1, a key gene in the PINK1‐PRKN‐dependent mitophagy pathway, impairing the ability of neurons to eliminate dysfunctional mitochondria and maintain mitochondrial homeostasis (Zhou et al. 2023). MiR‐494‐3p is particularly relevant due to its role in suppressing superoxide dismutase (SOD) activity, leading to increased oxidative stress, apoptosis, and mitochondrial dysfunction via the SIRT3 pathway in PD cell models (Geng et al. 2018). Additionally, miR‐320a‐3p has been implicated in modulating mitochondrial ROS production in neuronal and glial cells, further linking it to oxidative stress and PD‐related neurodegeneration (Shukla et al. 2023). MiR‐543‐3p and miR‐126‐5p are also highly relevant as they regulate SIRT1, a key factor in mitochondrial biogenesis and oxidative stress defense, suggesting their involvement in neuroprotection and disease progression in PD (Lee and Im 2021; Scheper et al. 2023).

To our knowledge, only a limited number of studies have explored the functions of miRNAs in relation to cognitive decline, symptom intensity, and overall QOL in individuals with Parkinson's disease (Da Silva et al. 2021; Han et al. 2020). Therefore we aimed to explore whether serum EV‐derived miRNAs involved in regulating oxidative stress and mitochondrial dysfunction (miR‐24‐3p, miR‐103a‐3p, miR‐320a‐3p, miR‐494‐3p, miR‐126‐5p, and miR‐543) are associated with the degree of motor impairment, the extent of cognitive decline, and patient‐reported QOL in PD. Our goal was to evaluate whether this panel of EV‐derived miRNAs could serve as accessible, biologically relevant indicators of disease burden, with the long‐term goal of enabling non‐invasive monitoring of disease progression and supporting timely, targeted clinical management.

## 2 Materials and Methods

### 2.1 Study Subject Selection

Adults diagnosed with PD were enrolled in this prospective observational cohort study at the Departments of Neurosurgery and Neurology at the Lithuanian University of Health Sciences Hospital in Kaunas, Lithuania.

Eligibility criteria included a confirmed idiopathic PD diagnosis with a good response to levodopa, no active or untreated psychiatric conditions, and no abnormalities detected on brain MRI. Exclusion criteria for the study included atypical Parkinsonism, dementia, and past or current psychiatric disorders.

Blood serum from 93 PD patients was collected during routine hospital visits. Cohort size was determined by feasibility and precedent from similar PD biomarker studies, where reported correlations were generally modest (Han et al. 2020; Wan et al. 2024). To support this, post hoc power analysis for this cohort was conducted using IBM SPSS Statistics (two‐tailed, *α* = 0.05) for both Pearson and Spearman correlations. The analysis indicated that the study had over 80% power to detect correlation coefficients (r/ρ) of approximately 0.30 and around 70% power for coefficients of 0.25.

A certified psychologist conducted neuropsychological tests, while clinical data were recorded by a neurologist and neurosurgeon.

The study was approved by the Kaunas Regional Biomedical Research Ethics Committee (BE‐2‐3, BE‐2‐48), and all participants gave written informed consent.

### 2.2 Psychological Assessment Procedures

A variety of neuropsychological tests were administered to comprehensively evaluate cognitive functioning in patients with PD. The assessment focused on multiple domains, including psychomotor speed, cognitive flexibility, verbal and non‐verbal fluency, cumulative learning, recognition, recall, and attention span. However, due to motor impairments and overall health conditions, completing a full neuropsychological evaluation was not always feasible.

The psychological assessments were conducted using the same methods described by Vaitkienė et al. (2024). Visual focus, psychomotor speed, mental flexibility, and other executive abilities were evaluated with the Trail Making Test (TMT). Verbal memory was assessed using the Rey Auditory Verbal Learning Test (RAVLT), while verbal executive function was measured with the verbal fluency test. The five‐point test was used to examine nonverbal executive function. Attention span, working memory, and psychomotor speed were assessed using the Wechsler Adult Intelligence Scale‐III (WAIS‐III). General cognitive impairment was calculated using the Mini‐Mental State Examination (MMSE) when the score was ≤ 25 and the Montreal Cognitive Assessment (MoCA) when the score was ≤ 20.

The Parkinson's Disease Questionnaire (PDQ‐39) was used to assess patients' quality of life (QOL) and the impact of Parkinson's disease symptoms.

### 2.3 Neurological Status Assessment

This study includes data from PD patients whose symptom severity was assessed using the Unified Parkinson's Disease Rating Scale (UPDRS) (Goetz et al. 2008). Each symptom was scored on a scale from 0 to 4, with 0 indicating no symptoms and 4 representing very severe symptoms. Additional information on the evaluated symptoms is provided in Table S1.

### 2.4 Sample Collection and Processing

Venous blood samples were collected from individuals with PD in coagulation‐activating tubes. Serum was separated from the blood by centrifugation at 3000 × *g* and stored at −80°C until the miRNA extraction step.

500 μL of serum was used for EV miRNA isolation using the Qiagen exoEasy Midi Kit (Cat. No. 76064) following the manufacturer's protocol. The kit enables direct isolation of EV‐associated miRNAs via membrane affinity columns that selectively capture EVs from serum while removing non‐vesicular components. This approach is consistent with MISEV2018 recommendations for pre‐clearing biofluids and using high‐purity EV enrichment methods, and is widely used in recent EV miRNA studies, including Kim et al. (2023) and Foley et al. (2024), which employed the same extraction protocol for serum‐derived EV RNA. Compared to ultracentrifugation, membrane‐affinity capture yielded greater EV‐derived RNA and was recommended as the method of choice for RNA studies (Gutiérrez García et al. 2020).

To monitor and standardize RNA extraction efficiency, 0.01 ng of synthetic phosphorylated cel‐miR‐39‐3p (Phos‐UCACCGGGUGUAAAUCAGCUUG) was added after the Quazol centrifugation step. The final elution was performed using RNase‐free water.

While the kit enriches miRNAs derived from EVs, it does not isolate intact vesicles; therefore, Qiagen XE buffer (Cat. No. 76214), which specifically purifies intact EVs, was additionally used for characterization. EV characterization was performed on aliquots from the same sample set (full dataset reported in a separate manuscript currently under review). Nanoparticle tracking analysis confirmed particles within the expected EV size range, and ELISA showed CD63 positivity with absence of APO‐A1, consistent with MISEV2018 guidelines. These findings support that the RNA extracted from these vesicles is EV‐derived.

For cDNA synthesis, 10 ng of extracted miRNA was used with the TaqMan Advanced miRNA cDNA Synthesis Kit (Cat. No. A28007), a widely used platform for small RNA profiling by other researchers (Dobricic et al. 2022; Fuentevilla‐Alvarez et al. 2024). The kit is specifically designed for small RNA cDNA synthesis and involves synthesis of 3′ poly(A) tail so oligo‐dT primer can attach to perform reverse transcription, as well as 5′ adaptor—so the miRNA amplification is performed. After reverse transcription, the kit uses a universal amplification step to uniformly increase the amount of cDNA for each miRNA. This ensures that even low‐expressing targets are detectable in qPCR, while preserving relative expression levels across samples.

### 2.5 Analysis of MiRNA Expression Levels

Real‐time qPCR (RT‐qPCR) was performed using the Thermo Fisher “TaqMan Fast Advanced Master Mix” and specific miRNA probes: hsa‐miR‐24‐3p (Assay ID: 477992_mir), hsa‐miR‐103a‐3p (Assay ID: 478253_mir), hsa‐miR‐320a‐3p (Assay ID: 478594_mir), hsa‐miR‐494‐3p (Assay ID: 478135_mir), hsa‐miR‐126‐5p (Assay ID: 477888_mir), hsa‐miR‐543 (Assay ID: 4778155_mir), and cel‐miR‐39‐3p (Assay ID: 478293_mir) for normalization. RT‐qPCR was conducted using a Quant Studio 3 Real‐time PCR system, under the following cycling conditions: denaturation at 95°C for 20 s, followed by 45 cycles of 95°C for 3 s and 60°C for 30 s. MiRNA expression levels were determined using the comparative −∆Ct method. For each sample, ΔCt was calculated as the mean raw Ct value of the target miRNA minus the mean raw Ct value of the reference miR‐39. Since a lower ΔCt indicates higher expression (and a higher ΔCt indicates lower expression), we multiplied all ΔCt values by −1 so that, in the graphs, larger values correspond to higher expression levels.

### 2.6 Statistical Methods and Data Processing

MiRNA expression was analyzed in relation to age using Pearson or Spearman correlation tests; comparisons between 2 groups were performed with either the Student's *t*‐test or Mann–Whitney *U* test, depending on data normality. Normality was assessed with the Kolmogorov–Smirnov test for groups larger than 50, and with the Shapiro–Wilk test otherwise; results are provided in Table S2. Associations between miRNAs and PD parameters were calculated with age included as a confounder for all variables except gender. Age adjustment was performed by residualizing miRNA expression on age via linear regression, then correlation of residuals was analyzed with each parameter. Analyses were conducted using IBM SPSS Statistics and GraphPad Prism, with statistical significance set at *p* < 0.05. No formal outlier tests were applied, and no data points or samples were excluded.

## 3 Results

### 3.1 Cohort Characteristics and miRNA Expression Analysis

In this analysis, blood serum samples from 93 patients with PD were examined to investigate the relationship between circulating extracellular vesicle (EV)‐derived microRNAs (miRNAs) and key clinical features of the disease. The number of participants per analysis varied (*n* = 55–93) due to incomplete clinical data or undetectable miRNA expression. For analyses with the smallest sample size (*n* = 55), the effect size detectable at 80% power was approximately *r* = 0.36. However, the correlations observed in this study (0.25–0.37) generally fell within the range detectable with adequate statistical power.

The average age of the patients in our cohort was 62 years, with ages ranging from 39 to 82. To investigate the impact of aging on miRNA expression, we examined age‐related changes and identified two miRNAs with significant associations: miR‐24‐3p levels declined with advancing age, while miR‐126‐5p levels increased. These findings are presented in Table 1.

**TABLE 1:** Relationship between miRNA and Parkinson's Disease patient age, illness duration, and other parameters.

		miR‐24‐3p	miR‐103a‐3p	miR‐320a‐3p	miR‐494‐3p	miR‐126‐5p	miR‐543
Age	Correlation coefficient	**−0.278** **	0.039	0.097	−0.105	**0.242** *	−0.140
	Sig. (2‐tailed)	**0.009**	0.714	0.364	0.360	**0.025**	0.206
	*N*	**87**	90	90	78	**85**	83
Age at symptoms onset	Correlation coefficient	−0.035	0.042	−0.050	0.048	0.004	0.021
	Sig. (2‐tailed)	0.748	0.693	0.639	0.674	0.973	0.850
	*N*	87	90	90	78	85	83
Illness duration	Correlation coefficient	0.011	−0.091	−0.005	0.044	−0.019	−0.039
	Sig. (2‐tailed)	0.920	0.395	0.962	0.699	0.863	0.726
	*N*	87	90	90	78	85	83
Gender (male vs. female)	Correlation coefficient	0.068	**0.203** *	0.143	0.043	**0.243** *	0.090
	Sig. (2‐tailed)	0.530	**0.045**	0.178	0.708	**0.025**	0.417
	*N*	87	**90**	90	78	**85**	83
Education	Correlation Coefficient	0.174	**0.291** **	**0.255** *	0.209	0.202	0.123
	Sig. (2‐tailed)	0.107	**0.005**	**0.015**	0.067	0.064	0.272
	*N*	87	**90**	**90**	78	85	83

*Note:* Associations between miRNA expression and patient data were evaluated, when miRNA expression was adjusted to age. A positive correlation coefficient indicates that miRNA expression increases with older age, later age at disease onset, longer disease duration, higher education level, and male gender. In contrast, a negative correlation coefficient suggests that miRNA expression decreases as these same parameters increase. Age and age at symptom onset for miR‐24‐3p, miR‐103a‐3p, miR‐126‐5p were calculated using Pearson‘s correlation, the rest—Spearman's correlation.

* Correlation is significant at the 0.05 level (2‐tailed).

** Correlation is significant at the 0.01 level (2‐tailed).

Given that PD predominantly affects older adults but can also begin at a younger age, we further explored the relationship between miRNA expression and age at disease onset. In our cohort, the age at onset ranged from 29 to 75 years, with a mean of 53 and disease duration ranged from 1 to 20 years (mean duration of 9 years). No observed significant differences were found with miRNA expression levels and disease onset or duration after adjusting to age as a confounder.

Then, based on findings by Castro‐Aldrete et al. (2025) suggesting gender may influence PD symptoms, we examined miRNA levels and found that males had higher levels of miR‐126‐5p and miR‐103a‐3p. However, symptom severity did not differ by gender in our cohort when evaluating 41 female and 52 male patients, indicating these differences may reflect biological variation rather than clinical presentation.

Additionally, since we had collected data on patients' education levels (45 patients with a university degree and 48 without a degree), we examined whether there was any link between education and miRNA expression. The results showed that patients with higher education had increased levels of miR‐103a‐3p and miR‐320a‐3p, representing a novel observation given the absence of prior evidence connecting these miRNAs to education. Additionally, miR‐494‐3p and miR‐126‐5p showed non‐significant trends, resulting in upregulation with higher education levels. As higher education has been associated with milder symptoms in neurodegenerative diseases like Alzheimer's (Sobral et al. 2015), it may also influence the course of PD—possibly through epigenetic mechanisms, cognitive reserve, or extranigral protective effect upon white matter integrity (Kotagal et al. 2015).

### 3.2 Association Between miRNA and Neurological Functioning

A reliable biomarker would greatly improve the speed and ease of assessing PD symptom severity. With this in mind, we investigated whether miRNAs associated with oxidative stress and mitochondrial dysfunction could help differentiate between levels of symptom severity and potentially serve as tools for clinical evaluation. To explore this, we analyzed the relationship between serum EV‐derived miRNAs and a range of neurological symptoms in PD patients, including bradykinesia, balance problems, gait issues, tremor, freezing, hallucinations, dyskinesia, as well as motor fluctuations (ON/OFF states) and nocturnal akinesia.

**FIGURE 1:**
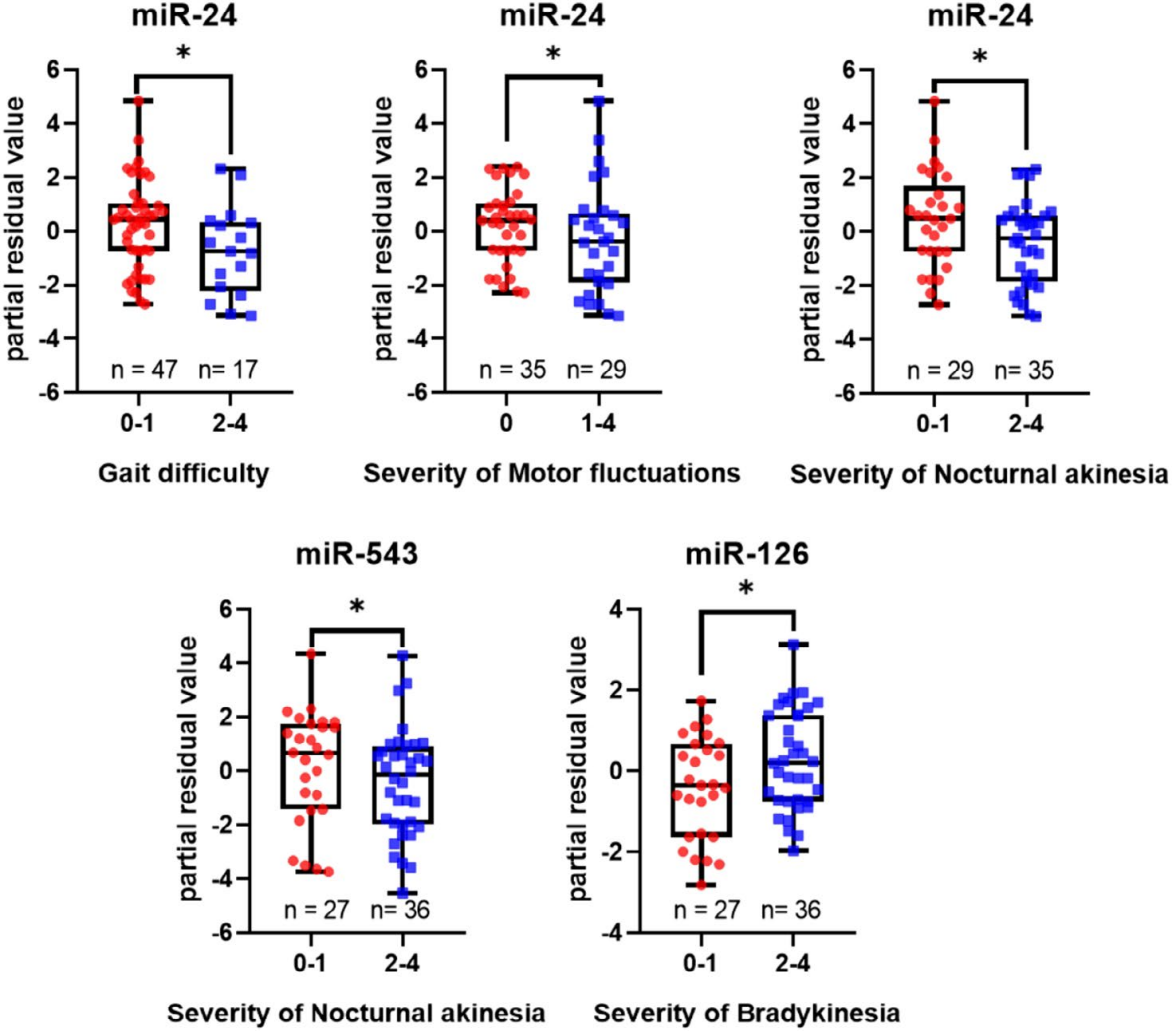
Association of miRNA expression with symptom severity. Each dot represents an individual miRNA expression value. Box plots show median (line), interquartile range (box), and minimum–maximum values (whiskers). Whiskers indicate the minimum and maximum expression levels, and the black line shows the median. Patients are grouped by motor symptom severity, indicated by the numbers below: 0—no symptoms, 1—low, 2—mild, 3—strong, 4—severe. *y* axis shows partial residual values when miRNA expression levels were adjusted to patients' age. Red dots represent patients with low symptom severity, while blue dots indicate those with more pronounced symptoms. Statistical significance between groups is indicated by * (*p* < 0.05). Student's *t* test was used for calculations. The sample sizes are mentioned in the graph as n. MiR‐24‐3p showed log_2_ fold change of −2.21 for gait impairment, −0.765 for motor fluctuations, and −1.33 for nocturnal akinesia. MiR‐543 exhibited an absolute log_2_ fold change of −1.07 with nocturnal akinesia, while miR‐126‐5p showed 1.145 with bradykinesia.

Results shown in Table 2 and illustrated in Figures 1 and 4, uncovered that elevated miR‐126‐5p levels were significantly associated with increased severity of bradykinesia, while reduced miR‐24‐3p levels were linked to more severe gait disturbances and motor fluctuations. Also, lower levels of miR‐543 and miR‐24‐3p were associated with more pronounced symptoms of nocturnal akinesia. To complement the statistical comparisons, we calculated log_2_ fold changes for these miRNAs and presented them in the legend of Figure 1.

**TABLE 2:** Relationship between miRNA and neurological functioning.

		miR‐24‐3p	miR‐103a‐3p	miR‐320a‐3p	miR‐494‐3p	miR‐126‐5p	miR‐543
Bradykinesia	Correlation coefficient	−0.188	−0.070	−0.037	−0.054	**0.254** *	0.060
	Sig. (2‐tailed)	0.138	0.581	0.767	0.685	**0.044**	0.640
	*N*	64	65	65	58	**63**	63
Tremor	Correlation coefficient	−0.173	−0.17	−0.210	−0.086	0.056	−0.093
	Sig. (2‐tailed)	0.173	0.175	0.093	0.522	0.663	0.470
	*N*	64	65	65	58	63	63
Gait	Correlation coefficient	**−0.266** *	−0.095	−0.091	−0.072	0.053	−0.154
	Sig. (2‐tailed)	**0.034**	0.452	0.470	0.593	0.681	0.227
	*N*	**64**	65	65	58	63	63
Balance	Correlation coefficient	−0.125	−0.098	0.039	0.036	0.084	−0.031
	Sig. (2‐tailed)	0.323	0.438	0.756	0.789	0.511	0.812
	*N*	64	65	65	58	63	63
Freezing	Correlation coefficient	−0.211	−0.010	−0.034	−0.044	−0.011	−0.219
	Sig. (2‐tailed)	0.094	0.938	0.788	0.743	0.932	0.085
	*N*	64	65	65	58	63	63
Hallucinations	Correlation coefficient	−0.227	−0.026	−0.092	−0.141	−0.064	−0.172
	Sig. (2‐tailed)	0.076	0.839	0.474	0.300	0.626	0.185
	*N*	62	63	63	56	61	61
Dyskinesias	Correlation coefficient	0.144	−0.009	−0.056	0.189	0.089	0.011
	Sig. (2‐tailed)	0.258	0.943	0.657	0.155	0.487	0.932
	*N*	64	65	65	58	63	63
On/off	Correlation coefficient	**−0.249** *	−0.198	−0.138	−0.192	0.045	−0.182
	Sig. (2‐tailed)	**0.047**	0.115	0.273	0.149	0.725	0.154
	*N*	64	65	65	58	63	63
Nocturnal akinesia	Correlation coefficient	**−0.266** *	−0.218	−0.134	−0.162	−0.202	**−0.255** *
	Sig. (2‐tailed)	**0.033**	0.082	0.286	0.224	0.112	**0.043**
	*N*	**64**	65	65	58	63	**63**

*Note:* Associations between miRNA expression and patient symptom severity were evaluated, when miRNA expression was adjusted to age. A positive correlation coefficient indicates that miRNA expression increases as symptoms become more severe. In contrast, a negative correlation coefficient suggests that miRNA expression decreases as the same parameters increase. Calculated using Spearman‘s correlation.

* Correlation is significant at the 0.05 level (2‐tailed).

Additionally, although not statistically significant, we observed a trend showing that reduced miR‐24‐3p levels were associated with more pronounced freezing and hallucinations, lower miR‐320a with tremor, decreased miR‐543 with freezing, and diminished miR‐103a‐3p with nocturnal akinesia. No correlations were found between the miRNAs and balance issues or dyskinesia.

These results suggest that miR‐24‐3p, miR‐126‐5p, and miR‐543 may play a role in the underlying pathological mechanisms of PD and could potentially serve as indicators of motor symptom progression.

A larger sample size could help confirm these trends or provide clearer insights into future research.

### 3.3 Correlation of miRNA With Cognitive Performance

To assess whether miRNAs in blood serum EVs could serve as potential biomarkers for monitoring cognitive function in PD, we examined their associations with various cognitive abilities that included psychomotor speed, mental flexibility, verbal and non‐verbal fluency, cumulative learning, recognition, recall, and attention span. A summary of the findings is provided in Table 3.

**TABLE 3:** Relationship between miRNA and cognitive functioning.

		miR‐24‐3p	miR‐103a‐3p	miR‐320a‐3p	miR‐494‐3p	miR‐126‐5p	miR‐543
Cognitive impairment (Mini‐Mental State Examination; The Montreal cognitive assessment)	Correlation coefficient	0.133	0.125	0.068	−0.044	**0.220** *	0.077
	Sig. (2‐tailed)	0.224	0.247	0.531	0.704	**0.046**	0.497
	*N*	85	88	88	76	**83**	81
Psychomotor speed (Trail making, Part A)	Correlation coefficient	−0.209	−0.177	−0.198	−0.13	**−0.314** **	−0.227
	Sig. (2‐tailed)	0.080	0.139	0.098	0.312	**0.009**	0.065
	*N*	71	71	71	62	**68**	68
Mental flexibility (Trail Making, Part B)	Correlation coefficient	−0.168	−0.057	0.069	−0.121	−0.149	−0.147
	Sig. (2‐tailed)	0.164	0.640	0.570	0.355	0.228	0.240
	*N*	70	70	70	61	67	67
Verbal fluency (Phonemic)	Correlation coefficient	−0.017	−0.135	−0.105	0.117	−0.11	−0.050
	Sig. (2‐tailed)	0.882	0.225	0.347	0.334	0.343	0.671
	*N*	79	82	82	70	77	75
Non‐verbal fluency	Correlation coefficient	−0.057	0.109	0.095	0.162	0.105	−0.030
	Sig. (2‐tailed)	0.618	0.331	0.401	0.185	0.367	0.802
	*N*	67	67	67	58	64	64
Attention span/working memory (Wechsler Adult Intelligence Scale‐III)	Correlation coefficient	−0.093	0.056	0.066	0	−0.066	0.001
	Sig. (2‐tailed)	0.415	0.617	0.558	0.999	0.566	0.996
	*N*	79	82	82	70	77	75
Psychomotor speed with learning (Wechsler Adult Intelligence Scale‐III, Digit Symbol Coding)	Correlation coefficient	−0.155	−0.034	−0.068	−0.126	−0.009	−0.202
	Sig. (2‐tailed)	0.227	0.794	0.594	0.360	0.946	0.125
	*N*	63	62	63	55	60	59
Delayed recall (Rey Auditory Verbal Learning Test, A7)	Correlation coefficient	−0.177	0.057	0.163	0.121	−0.033	−0.040
	Sig. (2‐tailed)	0.127	0.620	0.152	0.326	0.780	0.740
	*N*	77	79	79	68	74	73
Recognition (Rey auditory verbal learning test, recognition trial)	Correlation coefficient	−0.213	0.029	0.072	−0.038	−0.082	−0.062
	Sig. (2‐tailed)	0.063	0.799	0.529	0.758	0.490	0.603
	*N*	77	79	79	68	74	73

*Note:* Associations between miRNA expression and patient cognitive parameters were evaluated, when miRNA expression was adjusted to age. A positive correlation coefficient indicates that miRNA expression increases as tested parameters show more cognitive impairment. In contrast, a negative correlation coefficient suggests that miRNA expression decreases as the same parameters increase. Correlation between miR‐24‐3p, miR‐103a‐3p, miR‐126‐5p and delayed recall, non‐verbal fluency, verbal fluency were calculated using Pearson correlation; others with Spearman.

* Correlation is significant at the 0.05 level (2‐tailed).

** Correlation is significant at the 0.01 level (2‐tailed).

The results indicated that elevated miR‐126‐5p levels were linked to greater cognitive impairment. Furthermore, reduced expression of miR‐126‐5p was linked to slower psychomotor speed, as assessed by TMT Part A.

Also, though not statistically significant, low miR‐24‐3p and miR‐543 levels showed a subtle tendency with impaired psychomotor speed on TMT Part A test and low miR‐24‐3p with impaired recognition.

No associations were found regarding analyzed miRNA expression and mental flexibility, verbal and non‐verbal fluency, cumulative learning, delayed recall, and attention span.

**FIGURE 2:**
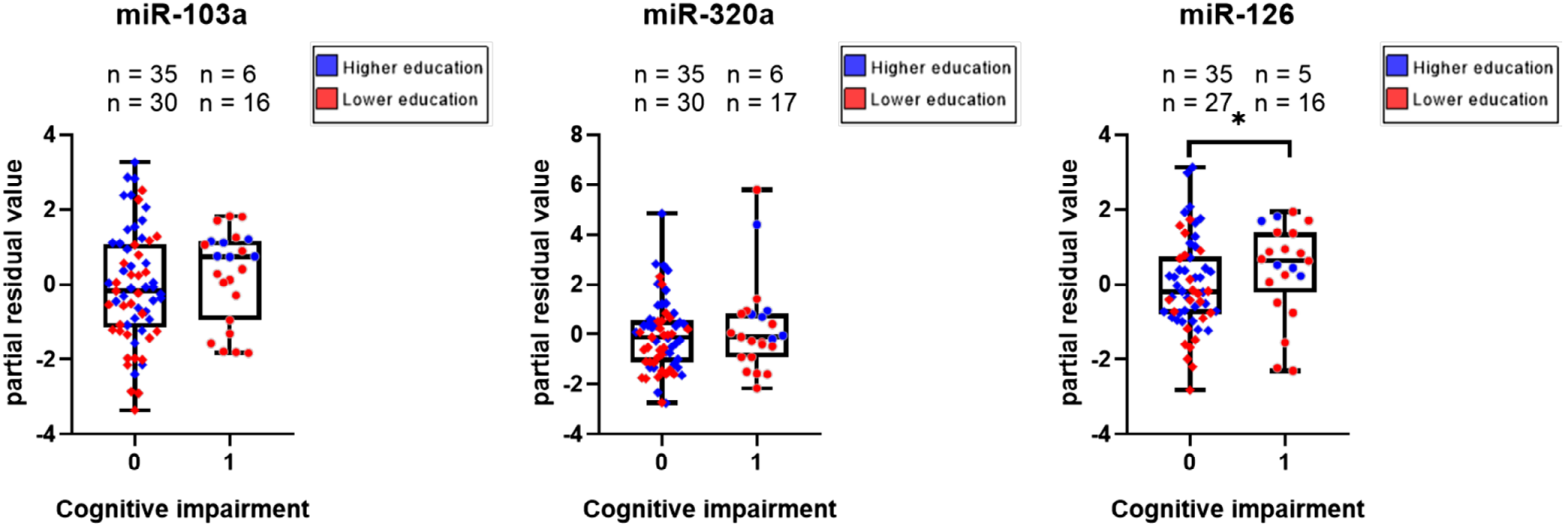
Association of miRNA expression with cognitive impairment and education. Each dot represents an individual miRNA expression value, with patients grouped according to cognitive status: 0 indicates normal cognition (Mini‐Mental State Examination > 25 or Montreal Cognitive Assessment > 20), and 1 indicates impaired cognition (Mini‐Mental State Examination ≤ 25 or Montreal Cognitive Assessment ≤ 20). *y* axis shows partial residual values when miRNA expression levels were adjusted to patients' age. Box plots show median (line), interquartile range (box), and minimum–maximum values (whiskers). Red dots represent patients with lower education, while blue dots correspond to those with higher education. Lower expression levels of miR‐126‐5p, miR‐320a‐3p, and miR‐103a‐3p among highly educated patients show less cognitive impairment. The third graph shows that elevated miR‐126‐5p levels are significantly linked to cognitive impairment between all patient groups (log_2_ fold change 0.5). Statistical significance between groups is marked with * (*p* < 0.05) when Mann–Whitney *U* test was used.

As previously noted, miR‐320a‐3p and miR‐103a‐3p levels were higher in individuals with greater educational attainment. To further investigate this, we examined whether the relationship between miRNA expression and cognitive impairment varied by education level. The analysis revealed a significant association between educational attainment and cognitive impairment in our cohort (*χ* ^2^ = 6.8; df = 1; *p* = 0.009), with only 14% of university‐educated individuals affected with cognitive impairment, compared to 38% among those without a university degree. Furthermore, Figure 2 illustrates that individuals with higher education exhibit less cognitive impairment when miR‐320a‐3p, miR‐103a, and miR‐126‐5p expression is reduced. These findings suggest a potential interaction between miRNA expression, educational level, and cognitive performance.

### 3.4 Impact of miRNA on Patient‐Reported Quality of Life

PD affects not only the physical health of patients but also their overall QOL (Al‐Khammash et al. 2023). To assess this impact, the PD Questionnaire (PDQ) was used to evaluate both symptom burden and QOL. We aimed to investigate whether specific miRNA expression patterns could help identify patients experiencing a decline in QOL. Such findings may aid in recognizing individuals who could benefit from additional support, such as assisted living services or social worker assistance for managing daily tasks.

**FIGURE 3:**
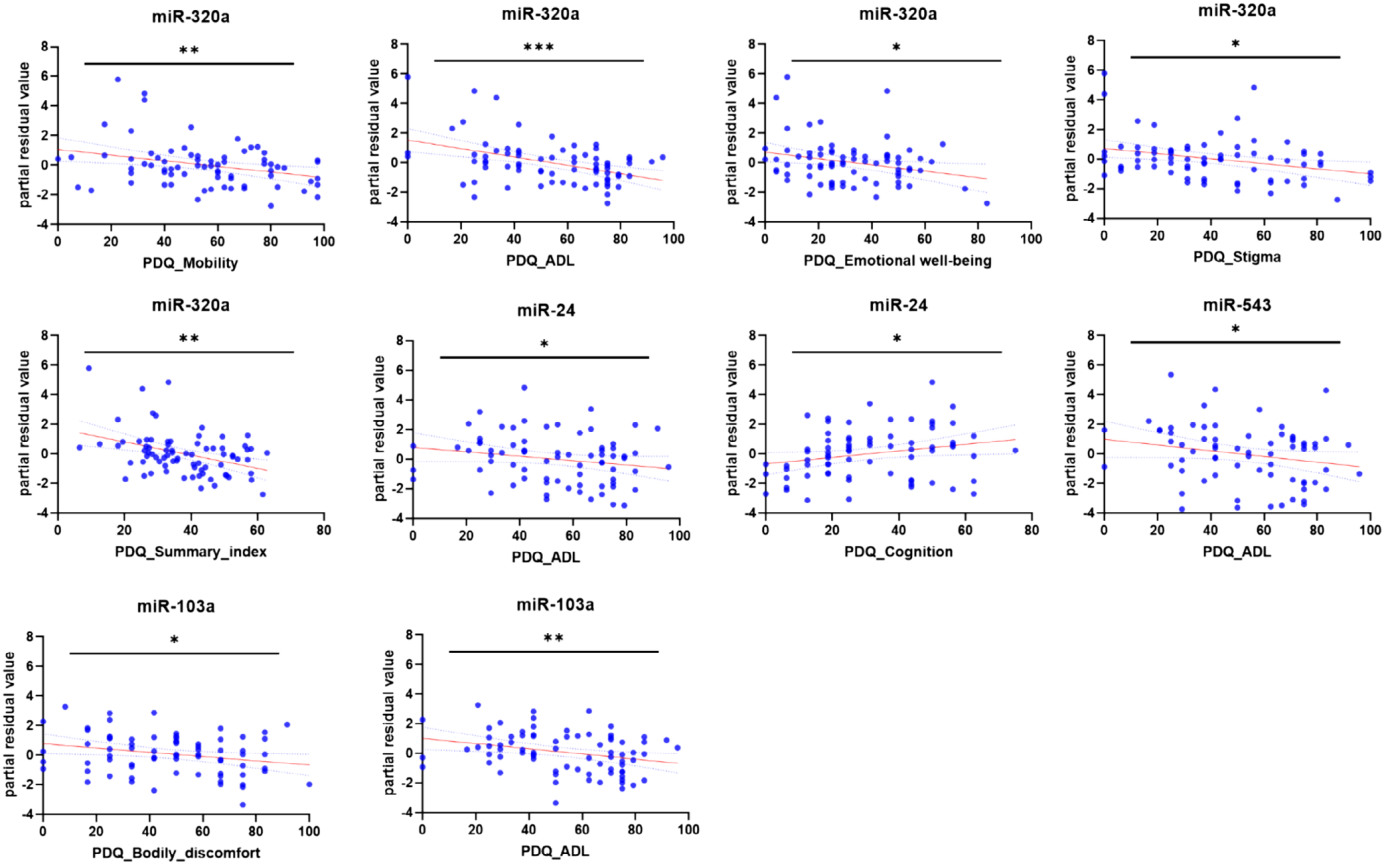
MiRNA expression and intensity motor, cognitive symptoms, and other parameters. Each dot represents an individual miRNA expression value, and the red line indicates the trend or correlation across the data points; the dotted line shows 95% confidence interval. Patients are grouped based on their Parkinson's disease questionnaire (PDQ‐39) questionnaire scores, which range from 0 (indicating optimal health) to 100 (indicating the most severe impairment) across domains such as mobility, Activities of Daily Living (ADL), stigma, social support, cognition, and overall quality of life (Summary index). The graph demonstrates that reduced expression of miR‐320a‐3p and elevated miR‐24‐3p levels are most consistently associated with worsening quality of life in Parkinson's Disease patients. Correlations were assessed using Pearson's test for miR‐103a‐3p and bodily discomfort, and Spearman's test for others. Sample sizes: MiR‐24‐3p (*n* = 74), miR‐320a‐3p (*n* = 78), miR‐103a‐3p (*n* = 77), miR‐543 (*n* = 74). *** Correlation is significant at the 0.001 level (2‐tailed). ** Correlation is significant at the 0.01 level (2‐tailed). * Correlation is significant at the 0.05 level (2‐tailed).

**FIGURE 4:**
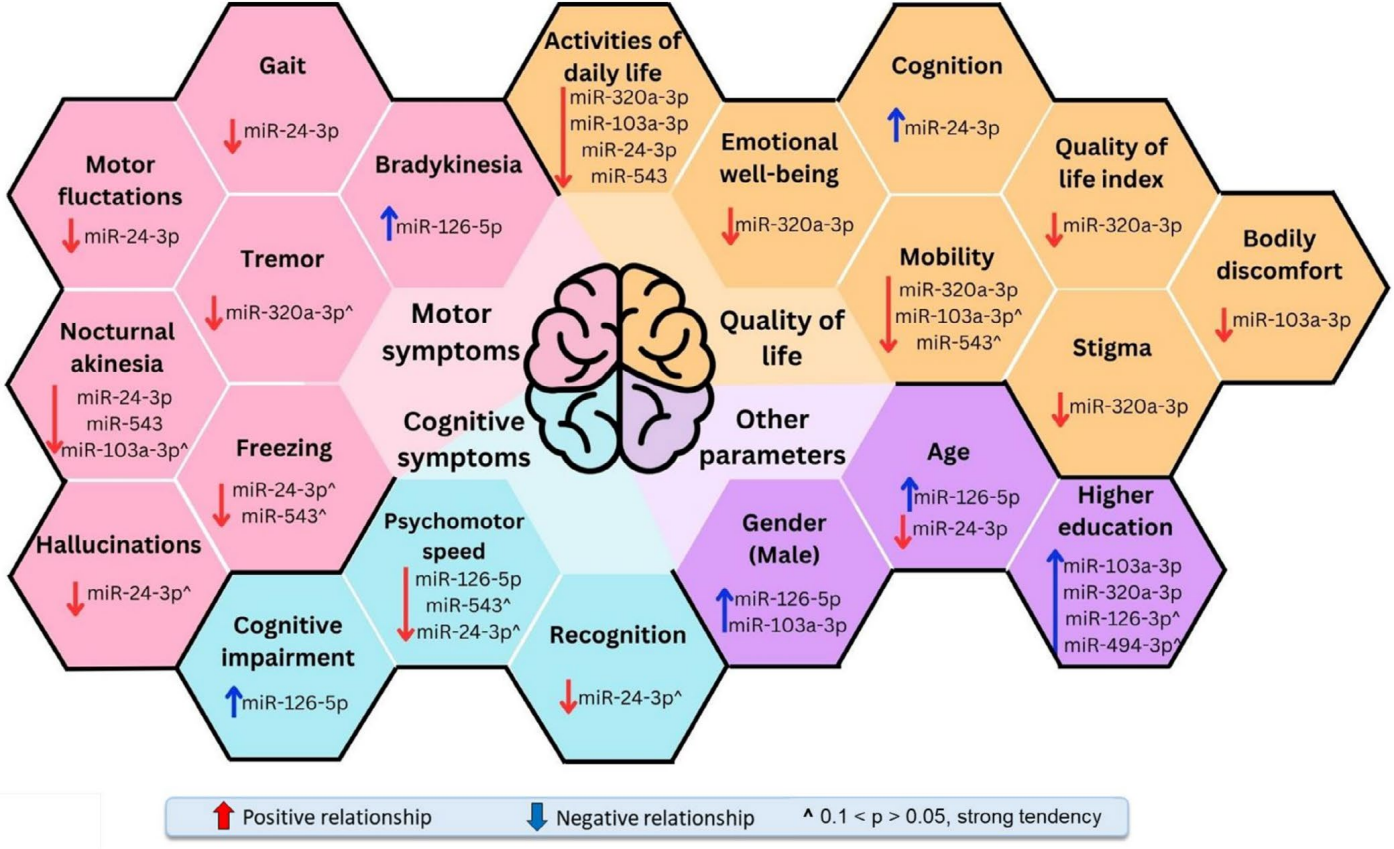
MiRNA association with Parkinson's Disease symptoms, cognitive, quality of life and clinical parameters. Figure depicting results from blood serum extracellular vesicles (EVs) of Parkinson's Disease patients. miRNA dynamics were analyzed in relation to the progression of motor and cognitive symptoms, as well as the worsening of quality of life parameters. Additionally, miRNA expression was examined concerning aging, disease duration, higher education levels, and male gender as a potential risk factor. Positive relationships show miRNA upregulation and negative relationships—downregulation. Additionally, ^ indicates observed trends between miRNA expression and clinical features that do not reach statistical significance but suggest a potential association.

Some reliable associations between miRNA levels and QOL indicators have been found. A summary of the findings is provided in Table 4 and illustrated in Figures 3 and 4.

**TABLE 4:** Relationship between miRNA and patient reported quality of life.

		miR‐24‐3p	miR‐103a‐3p	miR‐320a‐3p	miR‐494‐3p	miR‐126‐5p	miR‐543
Parkinson's Disease Questionare_Mobility	Correlation coefficient	−0.058	−0.209	**−0.286** *	−0.029	−0.051	−0.222
	Sig. (2‐tailed)	0.621	0.068	**0.012**	0.815	0.667	0.059
	*N*	74	77	**77**	70	74	74
Parkinson's Disease Questionare_Activities of daily living	Correlation coefficient	**−0.251** *	**−0.305** **	**−0.389** ***	−0.189	−0.143	**−0.249** *
	Sig. (2‐tailed)	**0.031**	**0.007**	**0**	0.117	0.224	**0.034**
	*N*	**74**	**77**	**77**	70	74	**74**
Parkinson's Disease Questionare_Emotional well‐being	Correlation coefficient	0.121	−0.118	**−0.226** *	0.093	−0.097	−0.087
	Sig. (2‐tailed)	0.306	0.307	**0.048**	0.443	0.413	0.467
	*N*	74	77	**77**	70	74	74
Parkinson's Disease Questionare_Stigma	Correlation coefficient	0.026	0.043	**−0.283** *	0.162	0.066	0.053
	Sig. (2‐tailed)	0.823	0.712	**0.012**	0.180	0.574	0.658
	*N*	74	77	**77**	70	74	74
Parkinson's Disease Questionare_Social_support	Correlation coefficient	0.212	0.123	−0.049	0.117	0.100	−0.024
	Sig. (2‐tailed)	0.070	0.288	0.674	0.335	0.398	0.842
	*N*	74	77	77	70	74	74
Parkinson's Disease Questionare_Cognition	Correlation coefficient	**0.263** *	0.160	0.006	0.034	0.136	0.120
	Sig. (2‐tailed)	**0.024**	0.165	0.959	0.778	0.248	0.314
	*N*	**74**	77	77	70	74	74
Parkinson's Disease Questionare_Communication	Correlation coefficient	0.185	0.043	−0.181	0.139	0.045	0.010
	Sig. (2‐tailed)	0.114	0.713	0.115	0.252	0.702	0.93
	*N*	74	77	77	70	74	74
Parkinson's Disease Questionare_Bodily_discomfort	Correlation coefficient	−0.182	**−0.255** *	−0.097	−0.116	−0.182	−0.131
	Sig. (2‐tailed)	0.121	**0.025**	0.401	0.337	0.121	0.269
	*N*	74	**77**	77	70	74	74
Parkinson's Disease Questionare_Summary_Index	Correlation coefficient	0.056	−0.120	**−0.335** **	0.005	−0.043	−0.145
	Sig. (2‐tailed)	0.635	0.300	**0.003**	0.969	0.713	0.220
	*N*	74	77	**77**	70	74	74

*Note:* Associations between miRNA expression and patient reported quality of life by Parkinson‘s Disease Questionare (PDQ‐39), when miRNA expression was adjusted to age. A positive correlation coefficient indicates that miRNA expression increases as tested parameters show declining quality of life parameters. In contrast, a negative correlation coefficient suggests that miRNA expression decreases as the same parameters increase. Correlations between miR‐24‐3p, miR‐103a‐3p, miR‐126‐5p and mobility, stigma, and summary index were calculated using Pearson correlation; others with Spearman.

* Correlation is significant at the 0.05 level (2‐tailed).

** Correlation is significant at the 0.01 level (2‐tailed).

*** Correlation is significant at the 0.001 level (2‐tailed).

Reduced miR‐320a‐3p expression was associated with greater mobility difficulties, increased challenges in daily activities, a stronger sense of stigma, worse emotional well‐being, and a lower overall quality‐of‐life index. Elevated miR‐24‐3p expression correlated with reduced self‐reported cognitive functioning. Conversely, decreased levels of miR‐24‐3p, miR‐543, and miR‐103a‐3p were linked to increased difficulties in daily activities, while lower miR‐103a expression was also associated with greater bodily discomfort.

Lower miR‐103a‐3p and miR‐543 levels showed a non‐significant trend toward greater mobility difficulties. Overall, miR‐320a‐3p expression consistently aligned with several indicators of declining quality of life, suggesting its potential relevance for further research.

## 4 Discussion

In this study, we investigated the association between serum EV‐derived miRNAs and clinical parameters related to PD, including intensity of symptoms, cognitive decline, and patient‐reported QOL. Specifically, we focused on miR‐24‐3p, miR‐103a‐3p, miR‐320a‐3p, miR‐494‐3p, miR‐126‐5p, and miR‐543, which have been implicated in the regulation of oxidative stress and mitochondrial dysfunction—two key pathological mechanisms in PD (Szelągowski and Kozakiewicz 2023).

Although PD is generally classified as an age‐related condition, the age at onset can differ significantly among patients, sometimes by several decades. Both aging and age‐related diseases can impact the motor system independently of PD. Moreover, advanced age has been linked to a more severe disease presentation and a faster rate of progression (Raket et al. 2022). Therefore, we aimed to determine whether certain molecular markers correlate with disease progression and symptom severity independent of age. Among the miRNAs analyzed, the strongest associations were observed with miR‐24‐3p and miR‐126‐5p.

It has been found that miR‐126‐5p acts as a key regulator of oxidative stress and mitochondrial dysfunction in PD, largely through its suppression of *SIRT1*, a critical factor in mitochondrial function and antioxidant defense. By downregulating *SIRT1*, miR‐126‐5p compromises neuroprotection and accelerates disease progression (Lee and Im 2021). Elevated miR‐126 expression has been observed in the dopaminergic neurons of the substantia nigra in PD patients and is linked to reduced neuronal survival and increased sensitivity of PD model cells (SH‐SY5Y) to the mitochondrial toxin 6‐OHDA (Kim et al. 2014). With aging, miR‐126‐5p levels naturally rise, further heightening neuronal vulnerability to stressors such as staurosporine and amyloid β1–42, both of which contribute to neurodegenerative processes. In contrast, miR‐126‐5p inhibition has shown neuroprotective effects against these stressors, emphasizing its role in regulating cellular resilience (Catanesi et al. 2020). At the molecular level, miR‐126‐5p interferes with the IGF‐1/PI3K/AKT signaling pathway, and its overexpression reduces AKT and ERK phosphorylation, thereby impairing stress responses and promoting mitochondrial dysfunction (Kim et al. 2014; Yang et al. 2018). Importantly, the results of our study also suggest that miR‐126‐5p upregulation may be associated with a distinct cognitive‐motor profile in PD, highlighting its potential as both a biomarker and therapeutic target. Our analysis revealed that higher EV‐derived miR‐126‐5p expression was associated with increased bradykinesia, cognitive decline but also with better psychomotor speed, suggesting a potential role in shaping a distinct cognitive–motor profile.

Additionally, miR‐126‐5p expression increased with patient age. However, without age‐matched controls, these associations may reflect aging‐related rather than PD‐specific processes. Oxidative stress is known to rise with aging even in healthy individuals and is further enhanced in PD, potentially becoming more pronounced when both factors interact (Kumar et al. 2012). Longitudinal studies will be important to clarify the role of miR‐126‐5p in disease progression.

Zhou et al. (2023) found that miR‐24‐3p regulates genes involved in mitochondrial function and cell survival by inhibiting PINK1, a key gene in the PINK1‐PRKN‐dependent mitophagy pathway, thereby impairing the ability of neurons to eliminate dysfunctional mitochondria and maintain mitochondrial homeostasis. Additionally, miR‐24‐3p is known to inhibit apoptosis by targeting pro‐apoptotic genes such as *BCL2L11* (BIM) and *FASLG* (Fas Ligand), both key players in programmed cell death (Regis et al. 2022). Higher levels may weaken this protective effect, potentially accelerating neuronal loss and disease progression (Zhou et al. 2023; Li et al. 2020). Another relevant target is ATP13A2, a lysosomal transmembrane P‐type ATPase involved in mitochondrial quality control. ATP13A2 deficiency causes impaired autophagy, oxidative stress, and accumulation of toxic proteins such as SNCA (Kett et al. 2015; Park et al. 2015; Gusdon et al. 2012) Notably, ATP13A2 expression is elevated in PD, while miR‐24‐3p is significantly downregulated, suggesting an inverse regulatory relationship (Ramirez et al. 2006; Yousefi et al. 2022). Our results demonstrate that miR‐24‐3p is progressively downregulated as motor symptoms worsen, suggesting that reduced levels of this miRNA may be detrimental to dopaminergic neurons. Notably, miR‐24‐3p expression was significantly lower in patients exhibiting more pronounced gait difficulties, motor fluctuations, and nocturnal akinesia, with a trend toward impaired recognition and psychomotor speed. Interestingly, an upregulation of miR‐24‐3p was linked to worsening in certain quality‐of‐life parameters, including impaired activities of daily living and self‐reported cognitive difficulties. From a demographic perspective, older patients also exhibited lower miR‐24‐3p expression levels.

The meta‐analysis by Schulz et al. (2019) represents one of the most comprehensive efforts to date to consolidate heterogeneous studies across brain, blood, and CSF, and it successfully identified several miRNAs with reproducible diagnostic differences between PD patients and controls, including upregulation of miR‐24‐3p in blood serum and miR‐126‐5p in brain tissue. In contrast, our study of serum extracellular vesicle miRNAs focused on within‐cohort associations, where we found that lower miR‐24‐3p and higher miR‐126‐5p levels correlated with worse symptom severity and cognitive decline. These differences may reflect the distinct biology of vesicle‐packaged versus total circulating miRNAs, but they also highlight that miRNA dynamics can differ when studied as diagnostic markers versus prognostic indicators. Together, our findings suggest that EV‐derived miRNAs may complement the diagnostic insights from Schulz et al. by capturing disease progression and symptom burden. Notably, both miR‐24‐3p and miR‐126‐5p have been linked to oxidative stress and mitochondrial dysfunction in PD, and our observed correlations with key motor and non‐motor symptoms such as bradykinesia, gait disturbances, and cognitive performance—support their potential relevance for refining clinical phenotyping and understanding disease heterogeneity (Zhou et al. 2023; Lee and Im 2021; Kim et al. 2014; Yang et al. 2018; Regis et al. 2022).

Geng et al. (2018) showed that miR‐494‐3p expression is increased and SIRT3 protein levels are decreased in MPP^+^‐induced SH‐SY5Y cells. Since SIRT3 protects neurons from metabolic and oxidative stress, its reduction leads to increased mitochondrial membrane permeability and cytochrome c–mediated apoptosis. Inhibition of miR‐494‐3p improved cell viability, enhanced SOD activity, and reduced apoptosis, caspase‐3, LDH, TNF‐α, IL‐1β, and ROS production in these cells (Cheng et al. 2016). In our study, miR‐494‐3p did not show correlations with motor symptoms, quality of life measures, or age‐related parameters.

Scheper et al. (2023) reported an upregulation of miR‐543 in the white matter of early‐stage PD patients, where it directly targets and downregulates SIRT1, a neuroprotective gene involved in mitigating oxidative stress and maintaining mitochondrial function, thereby contributing to early white matter changes in PD. Similarly, Wu et al. (2019) found that miR‐543 suppresses the expression and function of GLT‐1, a key glutamate transporter essential for glutamate clearance and neuronal protection; inhibition of miR‐543 was shown to restore GLT‐1 function and alleviate dyskinesia in PD models. Consistent with these findings, our study demonstrated a significant downregulation of miR‐543 in individuals experiencing more frequent episodes of nocturnal akinesia and greater impairment in activities of daily living, as well as a non‐significant trend toward impaired psychomotor speed and mobility.

Additionally, miR‐320a‐3p plays a role in PD by regulating autophagy and lysosomal pathways, targeting key genes like ATG5 to influence autophagic flux and the clearance of damaged proteins. It also modulates mitochondrial ROS production under stress conditions, impacting oxidative stress levels and neuronal survival. Additionally, miR‐320a‐3p promotes the release of exosomes that can transfer protective effects to neighboring cells by reducing mitochondrial ROS (Shukla et al. 2023). However, elevated miR‐320‐3p a levels can inhibit chaperone‐mediated autophagy by targeting HSP70, contributing to α‐synuclein accumulation and worsening mitochondrial dysfunction (Li et al. 2014). In our patient cohort, miR‐320a‐3p expression showed a stronger correlation with the non‐motor symptoms of PD. While it showed no significant relationship with core motor symptoms, lower miR‐320a‐3p levels were associated with reduced mobility and greater difficulty in performing daily activities. Furthermore, patients with lower miR‐320a‐3p expression reported a stronger sense of stigma, impaired mobility, activities of daily living, lower overall quality of life, and lower emotional well‐being. Also, an interesting pattern emerged in relation to cognitive function and education: in patients with higher levels of education, lower miR‐320a‐3p expression may be associated with reduced cognitive impairment, suggesting a potential interaction between miRNA expression and cognitive reserve mechanisms.

Lastly, Zhou et al. (2020) showed that miR‐103a‐3p is highly expressed in PD patient brain and plasma samples, as well as in MPTP‐induced mice and MPP^+^‐treated SH‐SY5Y cells, where it binds the 3′‐UTR of PRKN to downregulate Parkin and Ambra1, impairing mitophagy and leading to accumulation of dysfunctional mitochondria. This mitochondrial dysfunction elevated oxidative stress in dopaminergic neurons, exacerbating cell death. Inhibition of miR‐103a‐3p with antagomirs restores Parkin‐mediated mitophagy, reduces oxidative stress, enhances neuronal viability in vitro, and mitigates TH^+^ neuron loss and neurodegeneration in vivo. Our results on miR‐103a showed that lower expression was associated specifically with impaired activities of daily living and greater bodily discomfort. Nonetheless, there were some non‐significant trends suggesting links between lower expression and impaired mobility and nocturnal akinesia. Notably, as with miR‐320a‐3p, lower miR‐103a‐3p expression among highly educated individuals may also be associated with reduced cognitive impairment, pointing again to a possible interaction with cognitive reserve. These findings warrant further investigation to evaluate whether miRNAs might reflect mechanisms of cognitive resilience against impairment, especially since both education and cognitive performance in our cohort were significantly correlated. Previous studies have also found a negative correlation between years of education and UPDRS‐III motor scores, indicating that higher education levels are associated with better motor function. This implies that educational achievement may strengthen the brain's capacity to adapt to basal ganglia dysfunction, supporting both cognitive and motor resilience in PD—a concept known as motor reserve (Al‐Khammash et al. 2023; Blume et al. 2017).

Beyond miRNAs, several other fluid biomarkers have also been investigated in serum and plasma‐derived EVs from PD patients. Neuronally derived EV α‐synuclein species (total, oligomeric, and phosphorylated) have been associated with disease risk, phenoconversion, and motor severity (Yan et al. 2024; Chung et al. 2021). EV neurofilament light chain (NfL), a marker of neuroaxonal injury, has also been examined and shown promise as a progression marker in PD (Lin et al. 2019). Inflammatory processes are reflected in EV cargo as well, with α‐syn‐containing EVs capable of activating peripheral monocytes and stimulating cytokine release such as IL‐6 and TNF‐α (Liu et al. 2022). Importantly, miRNAs may intersect with these immune pathways: elevated miR‐21, miR‐155, and miR‐182 showed strong correlations with cytokine dysregulation in neurological disorders, including PD (Nikanfar et al. 2025). Together, these findings indicate that miRNAs should also be considered alongside other fluid biomarkers, as they may provide complementary information on clinical heterogeneity and disease progression in PD.

In conclusion, our findings highlight the potential of miRNAs as biomarkers for monitoring PD and guiding personalized treatment. While most studies focus on distinguishing PD from healthy individuals, we show that miRNA profiling may also reflect motor, cognitive, and quality‐of‐life symptoms. Understanding their role in neurodegeneration could open avenues for targeted therapies (Nies et al. 2021; Guévremont et al. 2023). However, several limitations exist. Firstly, the observed associations and group differences were modest in magnitude (correlation coefficients ~±0.2–0.39), yet statistically significant due to the consistency of the effects across participants. In complex, multifactorial conditions such as PD, small but reproducible molecular shifts are common and may still be biologically relevant, particularly for regulatory molecules like miRNAs that can exert amplified downstream effects. These findings should therefore be interpreted as preliminary and hypothesis‐generating, with their potential biological significance possibly lying in cumulative effects or in combination with other biomarkers. They warrant validation in larger, independent cohorts. Secondly, identified correlations between miRNA expression and clinical features cannot determine whether changes in miRNA levels directly cause these symptoms or if they are a consequence of disease progression. Third, measuring miRNAs in serum EVs may not fully capture brain changes, as miRNA levels can be influenced by external factors like comorbidities, medication, or lifestyle may also affect miRNA expression. It remains essential to determine whether these miRNAs are specific to PD by including healthy controls. Longitudinal studies tracking miRNA levels in the same individuals over time could reveal their relationship with treatment response and disease progression. Finally, combining miRNA data with neuroimaging or genetic markers may enhance diagnostic precision and support the development of precision medicine in PD (Azam et al. 2024).

## Author Contributions

**Violeta Belickienė:** methodology, writing – original draft, formal analysis, data curation, investigation. **Aistė Pranckevičienė:** conceptualization, formal analysis, data curation, investigation. **Andrius Radžiūnas:** conceptualization, data curation. **Andrėja Strigauskaitė:** methodology. **Ovidijus Laucius:** data curation. **Paulina Vaitkienė:** conceptualization, methodology, formal analysis, writing – review and editing, supervision, data curation, investigation.

## Funding

This research was funded by The Research Council of Lithuania, Grant S‐SEN‐20‐15, also by the Lithuanian University of Health Sciences fund.

## Ethics Statement

The research was carried out in alignment with the Declaration of Helsinki and received approval from the Kaunas Regional Biomedical Research Ethics Committee (No. BE‐2‐48, 19/05/2020).

## Consent

Informed consent was obtained from all subjects involved in the study.

## Conflicts of Interest

The authors declare no conflicts of interest.

## Supporting information


**Table S1:** Parkinson Disease symptom severity scoring according to Unified Parkinson's Disease Rating Scale (UPDRS).
**Table S2:** Normality test assessments between variables used for statistical analyses.

**Data S1:** jnc70355‐sup‐0002‐DataS1.xlsx.

## Data Availability

The data that supports the findings of this study are available in the Supporting Information of this article.
