# Supplementary material for: Oxidative Stress—Related Serum Extracellular Vesicle miRNAs Indicate Symptom Severity and Cognitive Decline in Parkinson's Disease
Source: J Neurochem. 2026 Jan 18;170(1):e70355. doi: 10.1111/jnc.70355 (PMC12813526; doi:10.1111/jnc.70355)
Supplement: Supplementary file 1 — Table S1: Parkinson Disease symptom severity scoring according to Unified Parkinson's Disease Rating Scale (UPDRS). Table S2: Normality test assessments between variables used for statistical analyses. [file JNC-170-0-s002.pdf]

Oxidative Stress – Related Serum Extracellular Vesicle miRNAs Indicate Symptom Severity and Cognitive Decline in Parkinson's disease

Violeta Belickienė[1], Aistė Pranckevičienė[2,3], Andrius Radžiūnas[4], Andrėja Strigauskaitė[1,6], Ovidijus Laucius[5], Paulina Vaitkienė[1]

SUPPLEMENTARY TABLES

Supplementary Table 1: Parkinson Disease symptom severity scoring according to Unified Parkinson's Disease Rating Scale (UPDRS).

**Symptom**	**Equivalent item at the Unified Parkinson's Disease Rating Scale**
Bradykinesia	Item 3.14 Global spontaneity of movement (body bradykinesia). This global rating combines all observations on slowness, hesitancy and small amplitude and poverty of movement in general, including a reduction of gesturing, and of crossing the leg.
Tremor	Item 3.18. Constancy of rest tremor. This item receives one rating for all rest tremor and focuses on the constancy of the rest tremor during the examination period when different body parts are variously at rest.
Gait	Item 3.10. Gait
Balance/Postural stability	Item 3.12 Postural stability
Freezing	Item 3.11. Freezing of gait
Nocturnal akinesia	Item 2.10 assesses difficulty turning in bed, affecting movement and sleep quality
Hallucinations or thought disorder (due to drug intoxication or dementia)	Item 1.2. Hallucinations and psychosis
Dyskinesia	Item 4.1. Time spent with dyskinesias
ON/OFF	Item 4.3: Time spent in the off state

Supplementary table 2. Normality test assessments between variables used for statistical analyses

miRNA	variable	Statistical test	statistic	p_value	df
mir-103a-3p	Age	Kolmogorov-Smirnov	0.04844	0.86871	90
mir-126-5p			0.05302	0.80454	85
mir-24-3p			0.06393	0.51296	87
mir-320a-3p			0.11047	0.00863	90
mir-494-3p			0.19303	1.2E-07	78
mir-543			0.10815	0.01897	82
mir-103a-3p	Disease duration	Kolmogorov-Smirnov	0.04844	0.86871	90
mir-126-5p			0.05302	0.80454	85
mir-24-3p			0.06393	0.51296	87
mir-320a-3p			0.11047	0.00863	90
mir-494-3p			0.19303	1.24E-07	78
mir-543			0.10815	0.01897	82
mir-103a-3p	Gender(man)	Kolmogorov-Smirnov	0.04844	0.86871	90
mir-126-5p			0.05302	0.80454	85
mir-24-3p			0.06393	0.51296	87
mir-320a-3p			0.11047	0.00863	90
mir-494-3p			0.19303	1.24E-07	78
mir-543			0.10815	0.01897	82
mir-103a-3p	Onset of the disease	Kolmogorov-Smirnov	0.04844	0.86871	90
mir-126-5p			0.05302	0.80454	85
mir-24-3p			0.06393	0.51296	87
mir-320a-3p			0.11047	0.00863	90
mir-494-3p			0.19303	1.24E-07	78
mir-543			0.10815	0.01897	82
mir-103a-3p	Education	Kolmogorov-Smirnov	0.04844	0.86871	90
mir-126-5p			0.05302	0.80454	85
mir-24-3p			0.06393	0.51296	87
mir-320a-3p			0.11047	0.00863	90
mir-494-3p			0.19303	1.24E-07	78
mir-543			0.10815	0.01897	82
mir-103a-3p	Attention span / working memory (Wechsler Adult Intelligence Scale III)	Kolmogorov-Smirnov	0.04983	0.88399	82
mir-126-5p			0.06389	0.61075	77
mir-24-3p			0.05897	0.71314	79
mir-320a-3p			0.10983	0.016	82
mir-494-3p			0.18915	1.50E-06	70
mir-543			0.10594	0.03886	74
mir-103a-3p	Cognitive impairment (Mini-Mental State Examination; The Montreal Cognitive Assessment)	Kolmogorov-Smirnov	0.04722	0.89917	88
mir-126-5p			0.04944	0.88545	83
mir-24-3p			0.05819	0.68013	85
mir-320a-3p			0.1127	0.00769	88
mir-494-3p			0.19794	7.77E-08	76
mir-543			0.10618	0.02618	80

mir-103a-3p	Delayed recall (Rey Auditory Verbal Learning Test, A7)	Kolmogorov-Smirnov	0.0466	0.94159	79
mir-126-5p			0.06044	0.72359	74
mir-24-3p			0.06281	0.63768	77
mir-320a-3p			0.11919	0.00738	79
mir-494-3p			0.18049	9.17E-06	68
mir-543			0.11118	0.02771	72
mir-103a-3p	Mental flexibility (Trail Making, Part B)	Kolmogorov-Smirnov	0.0621	0.72362	70
mir-126-5p			0.05075	0.93902	67
mir-24-3p			0.08134	0.30071	70
mir-320a-3p			0.13348	0.00347	70
mir-494-3p			0.18636	1.60E-05	61
mir-543			0.11243	0.03762	66
mir-103a-3p	Non-verbal fluency	Kolmogorov-Smirnov	0.04506	0.95202	81
mir-126-5p			0.05986	0.71872	76
mir-24-3p			0.06449	0.58578	78
mir-320a-3p			0.11065	0.01573	81
mir-494-3p			0.17446	1.90E-05	69
mir-543			0.11571	0.01687	73
mir-103a-3p	Verbal fluency (Phonemic)	Kolmogorov-Smirnov	0.03979	0.98776	82
mir-126-5p			0.04903	0.91902	77
mir-24-3p			0.06234	0.62959	79
mir-320a-3p			0.11013	0.0155	82
mir-494-3p			0.20537	8.69E-08	70
mir-543			0.1204	0.00975	74
mir-103a-3p	Psychomotor speed (Trail making, Part A)	Kolmogorov-Smirnov	0.05304	0.89061	71
mir-126-5p			0.05136	0.92805	68
mir-24-3p			0.07302	0.45841	71
mir-320a-3p			0.13611	0.00232	71
mir-494-3p			0.1861	1.35E-05	62
mir-543			0.09455	0.14491	67
mir-103a-3p	Psychomotor speed with learning (Wechsler Adult Intelligence Scale-III)	Kolmogorov-Smirnov	0.05764	0.8763	62
mir-126-5p			0.05934	0.86451	60
mir-24-3p			0.08907	0.24625	63
mir-320a-3p			0.12365	0.01794	63
mir-494-3p			0.18754	4.82E-05	55
mir-543			0.08618	0.33868	59
mir-103a-3p	Recognition (Rey Auditory Verbal Learning Test)	Kolmogorov-Smirnov	0.0466	0.94159	79
mir-126-5p			0.06044	0.72359	74
mir-24-3p			0.06281	0.63768	77
mir-320a-3p			0.11919	0.00738	79
mir-494-3p			0.18049	9.17E-06	68
mir-543			0.11118	0.02771	72
mir-103a-3p		Kolmogorov-Smirnov	0.05118	0.88724	77
mir-126-5p			0.05665	0.80648	74

mir-24-3p	Parkinson's Disease Questionare _Activities of Daily Living		0.06907	0.51621	74
mir-320a-3p			0.12723	0.00355	77
mir-494-3p			0.14829	0.0006	70
mir-543			0.11087	0.02662	73
mir-103a-3p	Parkinson's Disease Questionare _Bodily_discomfort	Kolmogorov-Smirnov	0.05118	0.88724	77
mir-126-5p			0.05665	0.80648	74
mir-24-3p			0.06907	0.51621	74
mir-320a-3p			0.12723	0.00355	77
mir-494-3p			0.14829	0.0006	70
mir-543			0.11087	0.02662	73
mir-103a-3p	Parkinson's Disease Questionare _Cognition	Kolmogorov-Smirnov	0.05118	0.88724	77
mir-126-5p			0.05665	0.80648	74
mir-24-3p			0.06907	0.51621	74
mir-320a-3p			0.12723	0.00355	77
mir-494-3p			0.14829	0.0006	70
mir-543			0.11087	0.02662	73
mir-103a-3p	Parkinson's Disease Questionare _Communication	Kolmogorov-Smirnov	0.05118	0.88724	77
mir-126-5p			0.05665	0.80648	74
mir-24-3p			0.06907	0.51621	74
mir-320a-3p			0.12723	0.00355	77
mir-494-3p			0.14829	0.0006	70
mir-543			0.11087	0.02662	73
mir-103a-3p	Parkinson's Disease Questionare _Emotional	Kolmogorov-Smirnov	0.05118	0.88724	77
mir-126-5p			0.05665	0.80648	74
mir-24-3p			0.06907	0.51621	74
mir-320a-3p			0.12723	0.00355	77
mir-494-3p			0.14829	0.0006	70
mir-543			0.11087	0.02662	73
mir-103a-3p	Parkinson's Disease Questionare _Mobility	Kolmogorov-Smirnov	0.05118	0.88724	77
mir-126-5p			0.05665	0.80648	74
mir-24-3p			0.06907	0.51621	74
mir-320a-3p			0.12723	0.00355	77
mir-494-3p			0.14829	0.0006	70
mir-543			0.11087	0.02662	73
mir-103a-3p	Parkinson's Disease Questionare _Social_support	Kolmogorov-Smirnov	0.05118	0.88724	77
mir-126-5p			0.05665	0.80648	74
mir-24-3p			0.06907	0.51621	74
mir-320a-3p			0.12723	0.00355	77
mir-494-3p			0.14829	0.0006	70
mir-543			0.11087	0.02662	73
mir-103a-3p	Parkinson's Disease Questionare _Stigma	Kolmogorov-Smirnov	0.05118	0.88724	77
mir-126-5p			0.05665	0.80648	74
mir-24-3p			0.06907	0.51621	74
mir-320a-3p			0.12723	0.00355	77
mir-494-3p			0.14829	0.0006	70

mir-543			0.11087	0.02662	73
mir-103a-3p	Parkinson‘s Disease Questionare _Summary_Index	Kolmogorov-Smirnov	0.05118	0.88724	77
mir-126-5p			0.05665	0.80648	74
mir-24-3p			0.06907	0.51621	74
mir-320a-3p			0.12723	0.00355	77
mir-494-3p			0.14829	0.0006	70
mir-543			0.11087	0.02662	73

Gait		Shapiro-Wilk		
		Statistic	df	Sig.
miR-24-3p	Group 0-1	0.973	47	0.346
	Group 2-4	0.927	17	0.193
Motor_fluctations		Shapiro-Wilk		
		Statistic	df	Sig.
miR-24-3p	Group 0	0.953	35	0.144
	Group 1-4	0.93	29	0.056
Nocturnal_akinesia		Shapiro-Wilk		
		Statistic	df	Sig.
miR-24-3p	Group 0-1	0.97	29	0.547
	Group 2-4	0.959	35	0.215
miR-543	Group 0-1	0.914	27	0.059
	Group 2-4	0.979	36	0.727
Bradykinesia		Shapiro-Wilk		
		Statistic	df	Sig.
miR-126-5p	Group 0-1	0.96	27	0.376
	Group 2-4	0.981	36	0.795

Cognitive impairment (Mini-Mental State Examination; The Montreal Cognitive Assessment)		Kolmogorov-Smirnov			Shapiro-Wilk		
		Statistic	df	Sig.	Statistic	df	Sig.
miR-126-5p	no_Cognitive Impairment	0.093	63	.200*	0.933	63	0.002
	Cognitive Impairment	0.154	21	.200*	0.918	21	0.081
miR-103a-3p	no_Cognitive Impairment	0.056	65	.200*	0.99	65	0.859
	Cognitive Impairment	0.159	23	0.138	0.897	23	0.062
miR-494-3p	no_Cognitive Impairment	0.186	56	0	0.934	56	0.004
	Cognitive Impairment	0.241	20	0.003	0.75	20	0

*. This is a lower bound of the true significance.
